# The Prevalence of Insomnia Among Patients With Chronic Tinnitus in the General Population of the Kingdom of Saudi Arabia

**DOI:** 10.7759/cureus.64295

**Published:** 2024-07-11

**Authors:** Almoaidbellah Rammal, Hussain Alsinni, Ameera A Alkhamesi, Ghada Alshahrani, Raghad N Bouges, Raghad Y Shosho, Manal O Aljuhani

**Affiliations:** 1 Otolaryngology - Head and Neck Surgery, King Abdulaziz University Faculty of Medicine, Jeddah, SAU; 2 Otolaryngology, Al-Jabr Eye, Nose, and Throat Hospital, Alahsa, SAU; 3 College of Medicine, King Abdulaziz University, Jeddah, SAU; 4 College of Medicine, Umm Al-Qura University, Makkah, SAU; 5 College of Medicine, Taif University, Taif, SAU

**Keywords:** sleep intiation, tinnitus, sleep disorder, insomnia, hearing

## Abstract

Background: Tinnitus is a perception of sound without external sound stimulation. Subjective tinnitus is the most common type and is unrelated to external sounds. It is a symptom, not an illness, and is often linked to various psychological factors like anxiety and depression. Insomnia is a personal sense of difficulty falling asleep and issues with sleep initiation, length, consolidation, or quality while having ample chance to sleep, which impairs one’s ability to function during the day. Sleep problems are prevalent in individuals with chronic tinnitus.

Objective: We aimed to assess insomnia prevalence in chronic tinnitus patients in Saudi Arabia.

Method: Our study, an online cross-sectional survey, included 434 Saudi participants with chronic insomnia, utilizing a Google Forms questionnaire (Google LLC, Mountain View, California, United States).

Results: A total of 434 participants responded to the online survey. The most represented age group was 18-25 years, and 319 (73.5%) of the respondents were female. Approximately one-third (34.6%, n=150) were from the southern region. In the sample, 184 (42.4%) participants had bilateral tinnitus, and 105 (24.2%) had had tinnitus for over two years. Around 62.7% of the participants suffered from insomnia due to tinnitus. In terms of sleep quality, 174 (40.1%) participants took over 40 minutes to fall asleep, 85 (19.5%) were often afraid to sleep due to disturbed sleep, and 63 (14.5%) frequently used sleep pills.

Conclusion: Our study of over 400 Saudi chronic tinnitus patients revealed that a large percentage of tinnitus patients have insomnia, influenced by geographic region and tinnitus duration. Our findings offer valuable insights, emphasizing the necessity for additional research to inform interventions aimed at enhancing the quality of life of individuals with chronic tinnitus.

## Introduction

Tinnitus is a perception of sound without an external sound stimulus. Subjective and objective tinnitus are the two main types of tinnitus [1]. The perception of acoustic vibratory activity produced mechanically within the body is called objective tinnitus. Structures in the circulatory, muscular, skeletal, or respiratory systems may be the source of objective tinnitus. Most individuals, however, experience subjective tinnitus, which is not connected to a specific sound source. This type of tinnitus is thought to be caused by or linked to damage to the auditory system [2].

Tinnitus is a symptom of several underlying conditions rather than a disease in itself. Presbycusis, otosclerosis, otitis, impacted cerumen, sudden deafness, Meniere’s disease, and other causes of hearing loss are otologic causes [3]. The stress felt by those with tinnitus has multiple dimensions and is exacerbated by distress cycles linked to psychological complexes other than tinnitus such as disorders of somatization, anxiety, depression, attention deficit hyperactivity disorder, and sleep [4]. Insomnia is described as a personal perception of trouble falling asleep and difficulty with sleep initiation, duration, consolidation, or quality despite having an excellent opportunity to sleep, and it affects the ability to function during the day [5,6].

Global tinnitus prevalence varies from 5% to 43%. Different types of tinnitus may be responsible for this variation. Although there is no data on the prevalence of tinnitus in Saudi Arabia, the prevalence of insomnia symptoms is estimated to be 30-35% worldwide based on epidemiological studies conducted in different countries [7,8].

In a 2015 study, researchers discovered that 76% of patients with tinnitus also experienced insomnia, indicating that individuals with chronic tinnitus frequently have sleep difficulties [9]. Another study conducted in 2023 found a positive correlation between the insomnia severity index and tinnitus handicap, with 72.2% of participants reporting poor sleep quality and 27.8% having moderate insomnia (r = 0.499, p = 0.035) [10]. This may have a detrimental impact on their daily activities and overall well-being. Therefore, it is crucial to investigate the relationship between sleep disorders and tinnitus, as one condition may exacerbate the other. Unfortunately, there is a scarcity of studies on this topic, particularly in the Kingdom of Saudi Arabia. Consequently, we conducted this study to measure the prevalence of insomnia among patients with chronic tinnitus in our region.

## Materials and methods

The present study was a cross-sectional investigation involving individuals in Saudi Arabia with chronic tinnitus. The study was approved by the Institutional Research Board at King Abdulaziz University, Jeddah, Saudi Arabia (approval number: HA-02-J-008). Participants were informed about the study’s objectives and participated voluntarily, providing informed consent before completing the questionnaire.

Participants were selected through convenience sampling. An Arabic version of the Tinnitus Handicap Inventory (THI) questionnaire, developed using Google Forms (Google LLC, Mountain View, California, United States), was distributed online via social media applications over two weeks (See Appendices). OpenEpi version 3.0 (www.openepi.com) was employed to determine the minimum required sample size, using a population estimate of 37,223,672 people (as reported by the General Authority of Statistics), with a 95% confidence interval (CI) and an expected frequency of 50%. The computed sample size was determined to be 385 participants, which was increased to 400 participants to account for potential data loss.

We used the Regensburg Insomnia Scale (RIS) to assess insomnia (See Appendices). The questionnaire is a comprehensive 10-item rating instrument that evaluates cognitive, behavioral, and emotional facets associated with psychophysiological insomnia. Each item was coded from 0 to 4. Therefore, the total score spans from 0 to 40 points, with higher scores correlating with heightened cognitive, behavioral, and emotional challenges characteristic of psychophysiological insomnia. Scores within the 0-12 range are deemed within the typical spectrum, while scores surpassing the established cutoff threshold (13 and above) signal the presence of symptoms consistent with psychophysiological insomnia, necessitating additional investigation.

Data analysis was conducted using RStudio (R version 4.3.1; R Foundation for Statistical Computing, Vienna, Austria). We used frequencies and percentages to express categorical variables. A Shapiro-Wilk test was applied to assess the normality of the RIS score, and a histogram was developed to depict the frequency distribution of the score. Factors associated with insomnia were evaluated using a univariable logistic regression analysis. The significantly associated variables in the univariable analysis were used as independent variables in a multivariable binary logistic regression model to determine the independent risk factors for insomnia. Results were presented in terms of odds ratios (OR) and 95%CIs, and statistical significance was considered to be p < 0.05.

## Results

Demographic characteristics

Approximately one-third (34.6%, n=149) of the participants were from the southern region, and 319 (73.5%) were female. Regarding age distribution, individuals aged 18-25 years represented the most prevalent group (37.6%, n=163). As for marital status, a majority of participants (52.3%, n=227) were married (Table 1).

**Table 1:** Demographic characteristics of the participants (N = 434) ^*^This variable had three missing records

Characteristic	Frequency (Percentage)
Region*	
Central	51 (11.8%)
Northern	58 (13.5%)
Southern	149 (34.6%)
Eastern	36 (8.4%)
Western	137 (31.8%)
Gender	
Male	115 (26.5%)
Female	319 (73.5%)
Age (years)	
18 to 25	163 (37.6%)
26 to 40	117 (27.0%)
41 to 55	108 (24.9%)
56 to 70	40 (9.2%)
71 to 85	6 (1.4%)
Marital status	
Single	175 (40.3%)
Married	227 (52.3%)
Divorced	18 (4.1%)
Widowed	14 (3.2%)

Characteristics of tinnitus

Of the total sample, 105 (24.2%) reported a duration of tinnitus perception exceeding two years. Additionally, 184 participants (42.4%) reported tinnitus affecting both ears bilaterally. Specifically, within this subgroup, the most frequently reported tinnitus characteristic was a whistling sound (66.4%, n=288). Furthermore, almost one-quarter (22.4%, n=97) of the respondents had trouble falling asleep at night because of tinnitus (Table 2).

**Table 2:** Characteristics of tinnitus (N = 434)

Characteristic	Frequency (Percentage)
Duration of tinnitus perception	
< 1 month	168 (38.7%)
1 to 6 months	59 (13.6%)
7 to 12 months	49 (11.3%)
1 to 2 years	53 (12.2%)
> 2 years	105 (24.2%)
Tinnitus laterality	
Right ear	138 (31.8%)
Left ear	112 (25.8%)
Bilaterally	184 (42.4%)
Tinnitus characteristics	
Whistling sound	288 (66.4%)
Hissing sound	65 (15.0%)
Waterfall sound	81 (18.7%)
Trouble falling asleep at night because of tinnitus	
No	162 (37.3%)
Sometimes	175 (40.3%)
Yes	97 (22.4%)

Participant responses to the RIS

Regarding poor sleep quality, less than half of respondents (40.1%, n=174) indicated that they took longer than 40 minutes to fall asleep, and 152 (35.0%) regularly slept for four hours or less. Additionally, 147 respondents (33.8%) said they did not sleep at night. Regarding sleep depth, a considerable proportion of participants mentioned that they mostly or always woke up early (56.0%, n=243), woke up from the slightest sound (42.6%, n=185), and had disturbed sleep (46.1%, n=200). Concerning the fearful focus on insomnia, 85 respondents (19.58%) reported that they were mostly or always afraid to go to bed because of disturbed sleep, and 186 (42.9%) that they thought about sleep. Finally, 63 (14.5%) of those with tinnitus took sleep pills mostly or permanently to get to sleep, while 157 (36.2%) indicated that they mostly or always felt in a state of health and well-being during the day (Table 3).

**Table 3:** Participants’ responses to the Regensburg Insomnia Scale (N = 434)

Characteristic	Frequency (Percentage)
Time needed to fall asleep (minutes)	
1 to 20	125 (28.8%)
21 to 40	135 (31.1%)
41 to 60	83 (19.1%)
61 to 90	39 (9.0%)
91 or more	52 (12.0%)
Sleep hours	
7 or more	112 (25.8%)
5 to 6	170 (39.2%)
4	87 (20.0%)
2 to 3	54 (12.4%)
0 to 1	11 (2.5%)
I wake up too early	
Never	20 (4.6%)
Seldom	47 (10.8%)
Sometimes	124 (28.6%)
Mostly	140 (32.3%)
Always	103 (23.7%)
I take sleeping pills in order to get to sleep	
Never	217 (50.0%)
Seldom	75 (17.3%)
Sometimes	79 (18.2%)
Mostly	37 (8.5%)
Always	26 (6.0%)
I am afraid to go to bed because of my disturbed sleep	
Never	100 (23.0%)
Seldom	97 (22.4%)
Sometimes	109 (25.1%)
Mostly	65 (15.0%)
Always	63 (14.5%)
I think a lot about my sleep	
Never	54 (12.4%)
Seldom	95 (21.9%)
Sometimes	99 (22.8%)
Mostly	78 (18.0%)
Always	108 (24.9%)
I feel that I have not slept all night	
Never	58 (13.4%)
Seldom	82 (18.9%)
Sometimes	147 (33.9%)
Mostly	77 (17.7%)
Always	70 (16.1%)
I wake up from the slightest sound	
Never	56 (12.9%)
Seldom	58 (13.4%)
Sometimes	135 (31.1%)
Mostly	79 (18.2%)
Always	106 (24.4%)
My sleep is disturbed	
Never	32 (7.4%)
Seldom	75 (17.3%)
Sometimes	127 (29.3%)
Mostly	84 (19.4%)
Always	116 (26.7%)
I feel fit during the day	
Always	39 (9.0%)
Mostly	118 (27.2%)
Sometimes	168 (38.7%)
Seldom	85 (19.6%)
Never	24 (5.5%)

RIS score and the prevalence of insomnia

**Figure 1:**
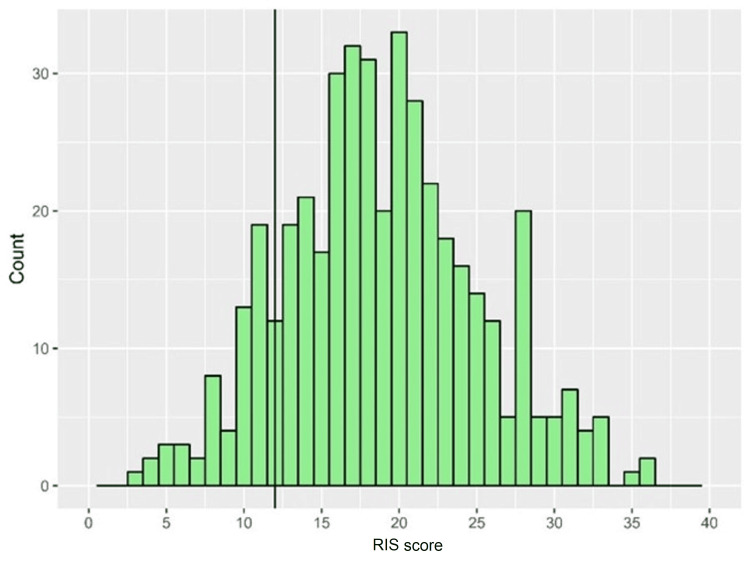
The distribution of RIS scores among participants, with the solid reference line indicating the cutoff threshold of insomnia. RIS: Regensburg Insomnia Scale

**Figure 2:**
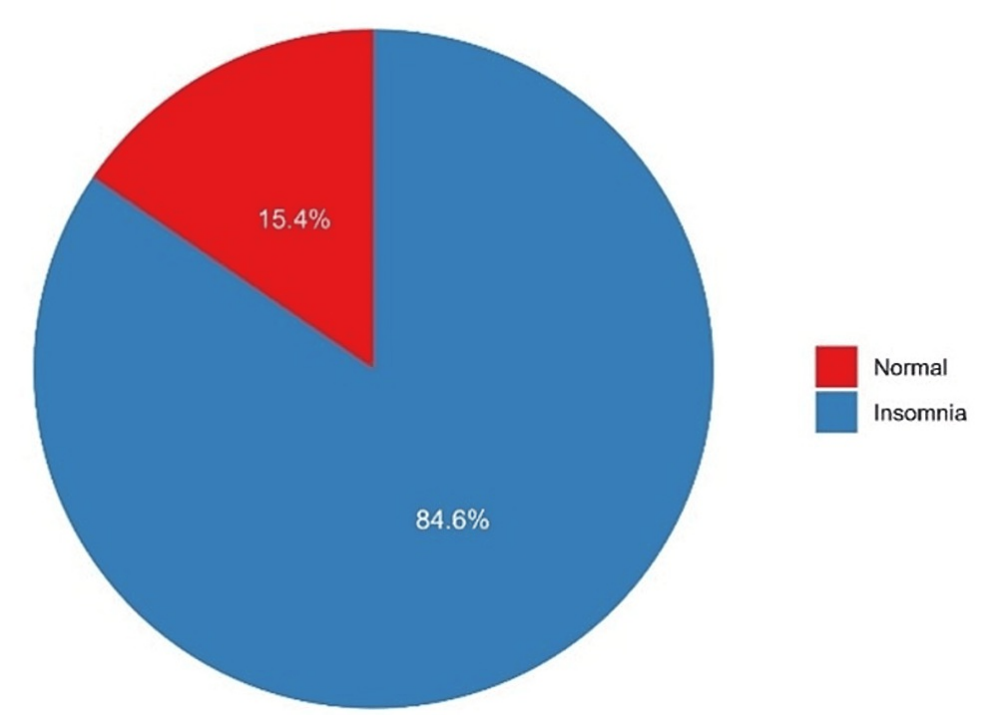
The prevalence of insomnia among study participants (N=434)

The median RIS score was 18.5 (interquartile range (IQR)=15-23), and the mean ± standard deviation (SD) score was 18.9±6.24. Assessment of normality according to the Shapiro-Wilk test showed that the RIS score was non-normally distributed (p=0.031), which was further confirmed by the visual depiction of the scores (Figure 1). The majority of participants had insomnia (84.6%, n=367), with an RIS score of 13 or higher (Figure 2).

Risk factors of insomnia among tinnitus patients

Among the risk factors examined for insomnia among the participating tinnitus patients, statistically significant associations were observed in the multivariable analysis. Notably, individuals in the northern region exhibited a significantly increased OR of 12.6 (95%CI 2.15-2.40; p=0.020), indicating a higher likelihood of experiencing insomnia compared to those in the central region, which served as the reference. Additionally, female participants had a significantly higher risk of insomnia compared to males, with an OR of 1.91 (95%CI 1.03-3.52; p=0.038). In terms of age, individuals in the age group of 56-70 years demonstrated a lower risk of insomnia, with an OR of 0.27 (95%CI 0.10-0.72; p=0.008). Furthermore, participants who reported having trouble falling asleep at night due to tinnitus had a substantially increased risk of insomnia relative to those who did not, with an OR of 4.75 (95%CI 1.98-13; p=0.001) (Table 4).

**Table 4:** Risk factors for insomnia among tinnitus patients (N=434) OR: odds ratio; CI: confidence interval

Characteristics	Univariable			Multivariable				
	OR	95% CI	p-value		OR	95% CI	p-value	
Region								
Central	Reference	Reference	-		Reference	Reference	-	
Northern	19.5	3.66, 362	0.005		12.6	2.15, 240	0.020	
Southern	1.48	0.68, 3.10	0.308		1.03	0.41, 2.43	0.951	
Eastern	3.76	1.10, 17.4	0.052		1.71	0.42, 8.75	0.474	
Western	1.79	0.81, 3.86	0.143		1.12	0.44, 2.72	0.810	
Gender								
Male	Reference	Reference	-		Reference	Reference	-	
Female	2.31	1.34, 3.96	0.002		1.91	1.03, 3.52	0.038	
Age								
18 to 25	Reference	Reference	-		Reference	Reference	-	
26 to 40	1.29	0.62, 2.82	0.502		0.99	0.45, 2.26	0.984	
41 to 55	0.69	0.35, 1.37	0.286		0.67	0.33, 1.39	0.280	
56 to 70	0.31	0.14, 0.70	0.004		0.27	0.10, 0.72	0.008	
71 to 85	0.30	0.05, 2.23	0.174		0.25	0.03, 2.20	0.168	
Marital status								
Single	Reference	Reference	-		-	-	-	
Married	0.72	0.41, 1.24	0.242		-	-	-	
Divorced	2.70	0.52, 49.8	0.345		-	-	-	
Widow	2.07	0.38, 38.4	0.494		-	-	-	
Duration of tinnitus perception								
< 1 month	Reference	Reference	-		Reference	Reference	-	
1 to 6 months	3.89	1.31, 16.7	0.030		3.85	1.24, 17.0	0.037	
7 to 12 months	1.25	0.54, 3.29	0.623		1.52	0.57, 4.50	0.420	
1 to 2 years	0.71	0.34, 1.56	0.381		0.84	0.37, 2.01	0.691	
> 2 years	1.16	0.60, 2.30	0.661		1.48	0.72, 3.14	0.298	
Tinnitus laterality								
Right ear	Reference	Reference	-		-	-	-	
Left ear	1.47	0.73, 3.07	0.286		-	-	-	
Bilaterally	1.13	0.62, 2.03	0.696		-	-	-	
Tinnitus characteristics								
Whistling sound	Reference	Reference	-		-	-	-	
Hissing sound	1.31	0.63, 2.98	0.494		-	-	-	
Waterfall sound	1.92	0.91, 4.54	0.107		-	-	-	
Have trouble falling to sleep at night because of tinnitus								
No	Reference	Reference	-		Reference	Reference	-	
Sometimes	1.52	0.87, 2.69	0.143		1.69	0.91, 3.20	0.098	
Yes	3.42	1.53, 8.71	0.005		4.75	1.98, 13.0	0.001	

## Discussion

Psychological and emotional distress are common in tinnitus patients, resulting in daily living changes, including sleeping disorders. Therefore, investigations should take place in patients reporting tinnitus to improve diagnosis, treatment, and quality of life [11]. 

We conducted a retrospective correlation analysis of 434 participants’ responses to the Tinnitus Questionnaire (TQ) and the RIS. Our findings show that the tinnitus patients in the study group not only had significant psychological symptoms associated with insomnia but also had disturbed sleep. In addition, these complaints were correlated with the severity of sleeplessness.

In our study, the most represented age group was 18-25 years (37.6%, n=163), and the majority of participants were female (73.5%, n=319) and married (52.3%, n=227). As far as tinnitus characteristics go, 42.4% (n=184) of the sample suffered from bilateral tinnitus, and 66.4% (n=288) from a whistling sound.

Most participants had tinnitus for over two years (24.2%, n=105) (Table 2). These data match the findings of a study conducted in Germany that showed that most patients were females with bilaterally affected ears [12]. Another study by Mantello et al. also found a predominance of bilateral tinnitus and female patients among participants [13]. On the other hand, another study showed that males were more likely to have tinnitus than females [14].

Nearly one-quarter of the participants in the present study had trouble falling asleep at night because of tinnitus, whereas a study by Koning showed that 50% of tinnitus patients develop sleep disability [15]. The anxiety brought on by tinnitus could probably intensify hyperarousal [14,16], which is significant in the development of insomnia [14,17]. More research is required to determine whether patients with tinnitus also experience hyperarousal symptoms similar to those of sleeplessness [18]. Assessing sleep quality according to the RIS score (Table 3), it was found that 40.1% (n=174) of the participants in the present study fell asleep within 40 minutes, and 35.0% (n=152) slept for four hours or less. In addition, most patients (56%, n=243) had poor overnight sleep and early morning wake-ups. These findings were similar to those of Attanasio et al., in whose study the tinnitus group had poorer sleep, shorter sleep times, and more frequent awakenings during the sleep cycle compared to a control group [19].

Our study showed a strong correlation between insomnia and tinnitus patients, as demonstrated by a median RIS score of 18.5 and a mean score of 18.9±6.24. There was a marked relationship between participants from the northern region of Saudi Arabia and insomnia and tinnitus, with an OR of 12.6 compared to the central region. Females and persons aged 56 and older also had a higher likelihood of developing insomnia. This association led 14.5% of tinnitus patients to take sleeping drugs. Those patients may have experienced insomnia because external sounds are diminished throughout the night, allowing the perception of tinnitus to increase and thus leading to poor sleep quality and anxiety [14]. Our findings add to a better understanding of the relationship between sleep and tinnitus by highlighting the emotional and cognitive factors that connect tinnitus and sleeplessness.

Lastly, it is worth noting two main limitations of the present study. First, self-report instruments, which have inherent limitations, were the source of all collected data. Consequently, it will be necessary to corroborate our findings in separate samples using different evaluation tools. Second, because the present study was cross-sectional, a direct interpretation is impossible. It will, therefore, be necessary to corroborate our findings in separate groups using different assessment tools and a longitudinal methodology. To the best of our knowledge, no valid study has been done in Saudi Arabia to measure the prevalence of insomnia in tinnitus patients. The current analysis could contribute to future scientific papers and initiatives on insomnia associated with tinnitus.

## Conclusions

Our study in Saudi Arabia focused on insomnia among chronic tinnitus patients, revealing a significant relation between the two conditions, but also a subjunctive report of the conditions that may affect it. Factors like age, gender, and location influence the likelihood of insomnia in tinnitus patients, with the northern region showing a notably higher incidence. Tinnitus characteristics exacerbate sleep disturbances, indicating a need for further research. Limitations of the study include reliance on self-reports and a cross-sectional design, necessitating future longitudinal studies for causality. More research is crucial for confirming our findings and understanding the complexities of tinnitus-related sleep issues. Our study underscores the importance of interventions to improve the quality of life for those experiencing tinnitus locally and globally, and it contributes valuable insights to the literature on tinnitus-related insomnia.

## Appendices

**Table 5:** Tinnitus Handicap Inventory (THI) questionnaire

	A	B	C	D	E
Duration of tinnitus prescription?	Less than a month	1-6 months	7-12 months	1-2 years	More than 2 years
Tinnitus laterality?	Bilaterally	Right ear	Left ear		
Tinnitus characteristics?	‘Whistling’ sound	‘Hissing’ sound	Waterfall sound		
Because to tinnitus do you have trouble falling to sleep at night?	Yes	No	Sometimes		

**Table 6:** Regensburg Insomnia Scale (RIS) questionnaire

Please rate the following questions for the past four weeks					
Q1	1-20 minutes	21-40 minutes	41-60 minutes	61-90 minutes	91 minutes or more
How many minutes do you need to fall asleep?	0	1	2	3	4
Q2	7 hours or more	5-6 hours	4 hours	2-3 hours	0-1 hour
How many hours do you sleep during the night	0	1	2	3	4
Q3: Rate the following statements	Never	Seldom	Sometimes	Mostly	Always
I wake up too early	0	1	2	3	4
I take sleeping pills in order to get to sleep	0	1	2	3	4
I am afraid to go to bed because of my disturbed sleep.	0	1	2	3	4
I think a lot about my sleep.	0	1	2	3	4
I feel that I have not slept all night.	0	1	2	3	4
I wake up from the slightest sound.	0	1	2	3	4
My sleep is disturbed.	0	1	2	3	4
I feel fit during the day	0	1	2	3	4
